# Transport Infrastructure, High-Quality Development and Industrial Pollution: Fresh Evidence from China

**DOI:** 10.3390/ijerph19159494

**Published:** 2022-08-02

**Authors:** Xiaole Wang, Feng Dong, Yuling Pan, Yajie Liu

**Affiliations:** 1 School of Economics and Management, China University of Mining and Technology, Xuzhou 221116, China; wxiaole1988@163.com (X.W.); ts19070137a31ld@cumt.edu.cn (Y.P.); tb18070005b2@cumt.edu.cn (Y.L.); 2 School of Business, Jiangsu College of Finance and Accounting, Lianyungang 222061, China; 3 Jiangsu Key Laboratory of Coal-Based Greenhouse Gas Control and Utilization, China University of Mining and Technology, Xuzhou 221008, China

**Keywords:** transport infrastructure, industrial pollution, high-quality development, industrial agglomeration

## Abstract

To achieve high-quality development, transport infrastructure will play a crucial role in China’s economic growth, but its damage to the ecological environment has not been paid enough attention. This study was based on panel data for 30 Chinese provinces for the period of 2004–2017. A comprehensive index system for high-quality development based on the new development concept was developed. This high-quality development index used the entropy weight method and integrated transport infrastructure, high-quality development, and industrial pollution into a comprehensive framework, and systematically examined the effects of transport infrastructure and high-quality development on industrial pollution emissions. It was found that transport infrastructure significantly contributed to industrial pollution emissions, and there was a regional heterogeneity and time lag, with high-quality development and industrial pollution having an inverted “U”-shaped relationship. Further analysis showed that transport infrastructure significantly affected high-quality development and industrial pollution through industrial agglomeration, reduced the inhibitory effect on high-quality development by promoting industrial agglomeration, and reduced industrial pollution emissions by promoting industrial agglomeration.

## 1. Introduction

Over the past 40 years of reform and opening up, China’s economy has continued to grow at a high rate, and the ratio of GDP to global warming potential (GWP) has increased from 2% at the beginning of the reform to 15% currently. During this period China has accounted for more than 30% of the world’s economic growth, and has become the second largest economy in the world [1]. Alongside China’s rapid economic growth, environmental pollution and the wasteful use of resources have become increasingly serious. According to China’s Ministry of Ecology and Environment, the quality of China’s natural environment has improved in the past three years, but it still faces difficulties and challenges. The most direct and effective way to improve the natural environment is to reduce the intensity of major pollutant emissions, which is a binding goal that has been included in China’s 12th, 13th, and 14th Five-Year Plans. While China’s economy continues to grow, industrial emissions have become an important source of environmental pollution, and a reduction in the intensity of pollution emissions per unit of output is essential to control industrial emissions [2]. Since the 1990s, the development of China’s transport infrastructure has continued to steadily develop, and road density has been gradually increasing at an average annual rate of 7.3% [3]. However, ecological damage is closely related to the continuous expansion of transport infrastructure, which has recently received general attention [4]. Large amounts of industrial emissions, wastewater, and smoke (dust) are generated during the construction and operation of transport infrastructure, and natural resources are also consumed, which have adverse impacts on local ecosystems. It is therefore necessary to consider how the development of transport infrastructure affects industrial pollution emissions and what intrinsic mechanisms are involved.

The deterioration of the natural environment has become an important social issue, and the Chinese government has raised environmental protection to an unprecedented level. This is directly related to the goal of achieving a beautiful China, and has become a major obstacle to China’s sustainable economic development. The 19th National Congress of the Communist Party of China proposed the “five-in-one” concept, including the construction of an ecological civilization, as the overall layout of socialism with Chinese characteristics. A consensus has been reached around the concept of green development as a way to promote the sustainable development of China’s economy, society, and natural environment. As a link between regional economic and social activities, the construction of transport infrastructure has realized the flow of production factors between regions and promoted the formation of regional economic integration, but it has also damaged the natural environment to a certain extent. The report of the 19th National Congress of the Communist Party of China states that China’s economy has shifted from a high-speed growth stage to high-quality development, and it is necessary to continuously strengthen the layout of the transport infrastructure network to achieve the goal of “a strong transportation country”. Studies have shown that the construction of transport infrastructure can increase pollution emissions, thereby inhibiting the quality of economic development and aggravating the deterioration of the natural environment [5]. In the new era, the Chinese government needs to consider the dialectical relationship between economic growth and transport infrastructure development, steadily promote economic and social transformation, upgrade transport infrastructure and ensure its rational layout, and enhance environmental quality and efficiency. High-quality development is a new development approach that integrates economic quantitative growth and improvements in quality, and is related to both improvements in the quality of the infrastructure network and the construction of an ecological civilization. There is a need to consider the impact of high-quality development on industrial pollution and how the construction of transport infrastructure will affect the quality of economic development. Resolving these issues would enable policy recommendations to be provided to the Chinese government to promote the layout of the transport infrastructure network and improve the natural environment in the new era.

Many researchers have focused on the relationship between transport infrastructure [6,7,8], economic quality development, and environmental pollution [9,10], and some valuable conclusions have been drawn. In recent years, China’s rapid economic development has resulted in serious environmental pollution. Transport infrastructure, a key factor supporting economic development, has often been studied for its impact on the quality of economic development and air pollution [11], but little research has been conducted on its impact on industrial pollution. With the introduction of the new development concept in China, the relationship between high-quality development and haze pollution has also been discussed [12], but its impact on industrial pollution has rarely been studied. Transport infrastructure development promotes economic development through industrial agglomeration [13], but its impacts on high-quality economic development and industrial pollution have not been fully studied. Therefore, this study aims to analyze the relationship between the role of transport infrastructure, high-quality development, and industrial pollution, with a focus on the impact of transport infrastructure through industrial agglomeration. This study makes the following four main contributions. First, transport infrastructure, high-quality development, and industrial pollution were integrated into a comprehensive framework to empirically test the effects of transport infrastructure and high-quality development on industrial pollution emissions at the regional level. This would provide a new perspective on how to build an ecological civilization. Second, a system to evaluate high-quality development was constructed based on the new development concept, and the entropy weight method was used to calculate the comprehensive score of high-quality development in each region, while the time-lagged effect of high-quality development on industrial pollution was also examined. Third, using industrial agglomeration as a mediating variable, this study further explored the mechanism by which transport infrastructure influenced high-quality development and industrial pollution. Fourth, given the uneven regional development, the heterogeneity of transport infrastructure and high-quality development on industrial pollution emissions was examined. This study provides practical recommendations not only for China’s economic and social transformation and quality benefits, but also for other countries that are in the process of promoting green transport infrastructure.

**Figure 1:**
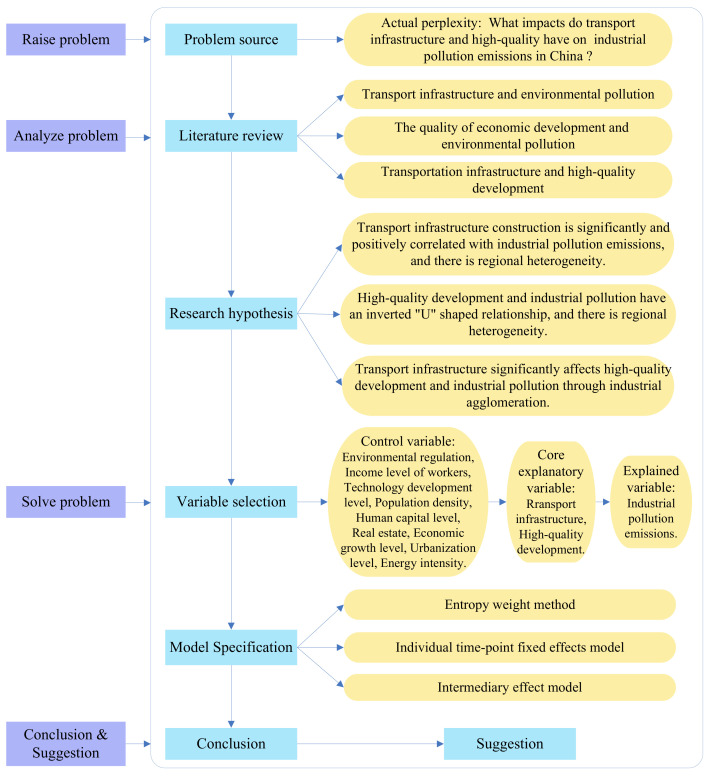
Analysis framework of the current study.

The remainder of this paper is structured as follows: Section 2 is a review of the relevant literature; Section 3 introduces the research hypotheses; Section 4 explains the research methodology, variables, and data collection; Section 5 represents the results and discusses the findings; Section 6 summarizes the findings and proposes policy recommendations. The specific idea is shown in Figure 1.

## 2. Literature Review

There is existing literature regarding the relationship between transport infrastructure and environmental pollution, but little research has been conducted on the influence of high-quality development on environmental pollution. There is currently a research focus on the environmental Kuznets curve (EKC), which describes the relationship between economic growth and environmental pollution. These studies have focused only on the single influence of quantitative economic growth on environmental pollution, while ignoring the important relationship between high-quality economic improvements and environmental pollution. Transport infrastructure is not only related to regional economic development, but also has a close link to environmental pollution.

Regarding research on transport infrastructure and environmental pollution, many scholars have demonstrated that transport infrastructure has significant negative impacts on the environment in terms of air pollution [6], traffic pollution [14], and carbon emissions [15,16]. One study also found an inverted “U”-shaped relationship between traffic density and urban smog in large and medium-sized cities, and direct emissions were an important channel through which traffic density affected smog formation [17]. In addition, there is also a close relationship between carbon emissions and energy efficiency. It has been shown that there is an intuitive network structure of carbon emissions across Chinese provinces [18], and transport infrastructure directly affects energy efficiency [19]. Although transport infrastructure contributes to regional economic growth and leads to industrial agglomeration, it will inevitably increase energy consumption and affect the natural environment [20]. Moreover, many researchers have also conducted studies on the environmental effects of transport infrastructure from different perspectives, such as the impact of urban road construction [21], the speed of transportation vehicles [22], and the number of vehicles on environmental pollution emissions [23].

To study the relationship between transport infrastructure and the quality of economic development, researchers have focused on three aspects: the promotional effect of transport infrastructure on economic development, the negative spillover effect on developed cities, and regional heterogeneity. Early research in this area was in the *American Scholar*, and one empirical study found that transport infrastructure and regional economic development are mutually reinforcing and co-developing [24]. Some researchers have also studied the performance of firms near high-speed railroads. It was found that on the Shinkansen extension in Japan, the opening of high-speed railroads improved the productivity of firms and increased their profits; thus, promoting economic development along the route [25]. In addition, transport infrastructure will also have an impact on employment and industrial specialization, which will lead to the development of the regional economy by reducing regional unemployment and increasing the number of employed people in relevant areas [26]. At the same time, the construction of transport infrastructure also consumes large amounts of resources, which leads to the reallocation of factor resources in upstream and downstream enterprises and expands the scope of labor mobility. This has an important impact on the production costs of enterprises in the region, especially the cost of human capital, which is conducive to improving the factor allocation efficiency of enterprises, and thus promoting economic development [27]. There are spillover effects of transport infrastructure because it facilitates economic growth and the spatial clustering of economic activities, and there are regional and industrial differences [28], as well as time-lag effects. With the implementation of China’s “One Belt, One Road” policy, the role of transport infrastructure connectivity has become more prominent [29], with asymmetric positive effects on countries and strong complementarity with trade facilitation and tariff reduction policies.

Regarding the study of economic development and environmental pollution, some scholars have focused on the EKC curve relationship between economic growth and environmental pollution, while less research has been conducted on how high-quality development affects environmental pollution. Many researchers have verified the existence of an EKC curve, with an inverted “U”-shape relationship between economic development and environmental pollution using data from different countries and regions [9,30], while clarifying the importance of economic and financial development on environmental performance [31]. In terms of the impact of environmental pollution on economic development, studies have found that environmental pollution significantly reduces urban employment and may also have significant hidden economic costs that inhibit economic development [32]. Some studies have found that increasing the tax rate on carbon emissions not only achieves a significant reduction in carbon emissions, but also saves operational costs; thus, promoting the coordination of ecological conservation and economic growth [33]. However, further analysis has found that managing the environment with a policy of licensing pollution through taxation inhibits regional economic development [34], and the higher the intensity of carbon emissions, the greater the welfare loss [10]. It has also been found that attracting foreign direct investment enhances capital accumulation, which generates environmental pollution, and when the inflow of foreign direct investment is restricted, economic growth is inhibited; although, environmental quality is improved [35].

Although there are extensive studies on the relationships between transport infrastructure, economic growth, and environmental pollution, research on the impacts of transport infrastructure and high-quality development on industrial pollution is extremely scarce. There are many factors and complex influential mechanisms affecting the impacts of transport infrastructure and high-quality development on industrial pollution, and there is an urgent need to clarify the internal connections and relationships between them. Such studies will enable the relationship between reform and development and the promotion of high-quality development to be better understood and managed. Therefore, this study developed a theoretical model to analyze the mechanism of the interaction between transport infrastructure, high-quality development, and industrial pollution.

## 3. Research Hypotheses

Since China’s reform and opening, many regions have embraced the simple idea of “build roads to get rich”, and the construction of transport infrastructure has steadily advanced, ensuring the continuous expansion of road mileage and a steady improvement in road grades, which has effectively boosted and guaranteed China’s continuous rapid economic growth [36,37]. Although the construction of transport infrastructure drives regional economic growth, it also damages the natural environment to some extent [3,20]. Many studies have found that transport infrastructure has an impact on air pollution through changes in transportation modes [21,26], and an increase in road length will lead to more vehicles, which will increase air pollutant emissions [28]. It has also been found that the improvement of transport infrastructure triggers the inter-regional movement of elements, which has an impact on environmental pollution [5]. Although many researchers have found that the construction of transport infrastructure has a significant negative impact on environmental pollution (air pollution, carbon emissions, traffic pollution, etc.) [6,14,15,16], there have been fewer studies on the impact of regional industrial pollution. The improvement in transport infrastructure has accelerated China’s industrialization, and industrialization has driven rapid economic growth, but this has been accompanied by serious industrial pollution [38]. Analyzing the relationship between industrial pollution and transport infrastructure will help the optimization of transport network layout and the formulation of environmental policies. In addition, given the differences in transport infrastructure construction between regions, this will likely directly determine the impact of transport infrastructure on industrial pollution [39]. Based on this, the first hypothesis was proposed.

**H1.**  *Transport infrastructure construction is significantly and positively correlated with industrial pollution emissions, and has regional heterogeneity*.

In recent years, the quality of China’s natural environment has improved, but the situation is still not satisfactory. How to reduce the intensity of industrial pollution emissions and break through the “zero-sum” thinking of high-quality development and environmental protection is an important issue that needs to be resolved in the current economic transformation context to ensure the coordination of economic growth and environmental quality, and thus achieve high-quality economic growth. High-quality development not only helps promote sustainable economic development, but also helps to curb environmental pollution emissions and improve the natural environment. According to the traditional EKC curve theory, after achieving a certain level of economic development, environmental pollution may reduce as the economy continues to develop [40]. The fundamental purpose of economic development is to achieve high-quality development. The study of the EKC curve relationship between economic development and environmental pollution has been discussed in depth by academics, such as the inverted U-shaped relationship [41]. Since the concept of high-quality economic development was introduced at the 19th People’s Congress of China, the connotation of economic development has become increasingly clear and its impact on environmental pollution has become an important issue. The core of high-quality economic development lies in changing economic growth dynamics, optimizing industrial structures and upgrading technology, and adhering to high-quality economic development helps to bring into play the structural and technological effects of economic development on environmental pollution [12]. With the continuous improvement of the quality of economic development, the coupling between industrial structure and economic development will change, and the improvement of transportation infrastructure can play a positive role in promoting industrial agglomeration and development [11]. Moreover, in order to reduce environmental pollution, the government is more willing to increase R&D investment and introduce advanced production technology to develop the economy, which not only improves the efficiency of resource use, but also effectively increases the reduction in pollution emissions [42]. Therefore, it is important to realize that the relationship between high-quality development and industrial pollution is similar to the traditional EKC curve. At the same time, it is also important to realize that the problem of unbalanced and insufficient economic and social development in China is extremely prominent [43], and the impact of high-quality development on industrial pollution may not be the same in different regions. Based on this, the second hypothesis was proposed.

**H2.**  *High-quality development and industrial pollution have an inverted “U”-shaped relationship, and have regional heterogeneity*.

In the new era of high-quality development, achieving advances in the scale and quality of the transportation network is important to serve and support the sustained, rapid, and healthy development of the economy and society. To improve the natural environment, the following should be addressed: adhere to supply-side reform as the main outcome; reform and innovate as the fundamental driving forces; coordinate development and safety; adhere to the principles of integrated, safe, innovative, efficient, and green development; accelerate the construction of a strong transportation country; and promote the high-quality development of China’s economy. Pollution emissions continuously accumulate during unplanned economic growth and continuously reduce the environmental carrying capacity and ecological quality of society [44]. Once the accumulated pollutants exceed the self-purification capacity of the environment, there will inevitably be irreversible consequences such as the destruction of ecological balance and deterioration of environmental quality. The construction of transport infrastructure helps to increase regional economic growth, reduce income inequality and energy poverty [45,46], and improve the productivity of enterprises through industrial agglomeration [47,48]. This will promote economic development; although, the impact on the quality of regional economic development is unknown. Many studies have shown that industrial agglomeration is a key channel through which transport infrastructure affects environmental pollution [49,50], but its relationship is uncertain. On the one hand, industrial agglomeration inevitably increases energy consumption, causing damage to the local natural environment [20,51]; on the other hand, industrial agglomeration will improve the level of production technology and management efficiency, improving energy efficiency and effectively alleviating the pressure on the local natural environment [43,52]. Given that the important role of industrial agglomeration on the quality of regional economic development and industrial pollution is uncertain, the third hypothesis is proposed.

**H3.**  *Transport infrastructure significantly affects high-quality development and industrial pollution through industrial agglomeration*.

**Figure 2:**
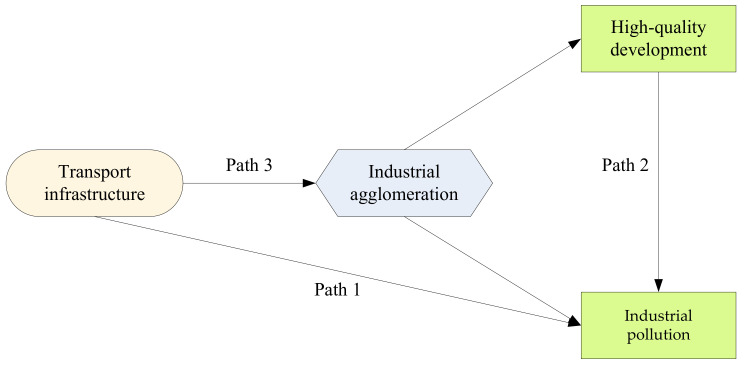
Mechanism analysis.

The global mechanism analysis is illustrated in Figure 2.

## 4. Methods, Variables, and Data

### 4.1. Model Specification

To examine the impact of transport infrastructure on industrial pollution emissions in China, the following benchmark regression model was constructed:

(1) $${\mathrm{SO}}_{2it}=\alpha +\beta TRA_{it}+\gamma Z_{it}+\mu_{i}+\eta_{t}+\varepsilon_{it}$$

where *i* and *t* represent province and year, respectively; SO_2_ denotes industrial pollution emission intensity; TRA denotes a transport infrastructure variable, whose coefficient *β* measures the impact of transport infrastructure on industrial pollution emissions and is therefore the core parameter of interest in this study; *Z* is a series of control variables; *γ* is the matrix of marginal impact coefficients of control variables; $\mu_{i}$ is individual fixed effect; $\eta_{t}$ is time fixed effect; and $\varepsilon_{it}$ is a random error term. By controlling for double fixed effects, the effects of regional heterogeneity and macro shocks that did not vary with time were eliminated.

On this basis, we were able to explore the heterogeneous impact of high-quality development in the construction of transport infrastructure on industrial pollution reduction in China. This enabled an analysis of the synergistic impact of both transport infrastructure and high-quality development on industrial pollution reduction in China. According to the traditional EKC curve theory, after a certain level of economic development is achieved, environmental pollution may be reduced as the economy continues to develop. Therefore, we added a quadratic high-quality development term (*HQD*) to the model for a regression analysis to investigate whether the relationship between industrial pollution and high-quality development was similar to that described by the traditional EKC curve. Therefore, we used the *HQD*, a composite indicator of high-quality development in each region, and the transport infrastructure variable *TRA* and its quadratic term in the study, which were added to Equation (1) to obtain:

(2) $${\mathrm{SO}}_{2it}=\alpha +\beta_{1}TRA_{it}+\beta_{2}HQD_{it}+\beta_{3}HQ{D_{it}}^{2}+\gamma Z_{it}+\mu_{i}+\eta_{t}+\varepsilon_{it}$$

where $\beta_{1}$, $\beta_{2}$, and $\beta_{3}$ are the core explanatory variable elasticity coefficients, respectively.

Furthermore, this study examined the internal mechanism by which transport infrastructure affects high-quality development and industrial pollution, and constructed a mediating effect model, with industrial agglomeration as a mediating variable, as follows:

(3) $$AGG_{it}=\alpha +\beta TRA_{it}+\gamma Z_{it}+\mu_{i}+\eta_{t}+\varepsilon_{it}$$

(4) $$HQD_{it}=\alpha +\beta TRA_{it}+\sigma AGG_{it}+\gamma Z_{it}+\mu_{i}+\eta_{t}+\varepsilon_{it}$$

(5) $$SO_{2it}=\alpha +\beta TRA_{it}+\sigma AGG_{it}+\gamma Z_{it}+\mu_{i}+\eta_{t}+\varepsilon_{it}$$

where $AGG_{it}$ denotes the industrial agglomeration level of Chinese province *i* in year *t*. The other variables are defined as described previously.

### 4.2. Variables

#### 4.2.1. Transport Infrastructure

Considering that the cost of air transport is extremely high and pipelines are not the main mode of transportation for industry, roads, railroads, and inland waterways were selected as proxy variables for transport infrastructure. Referring to Sun et al. (2019) and Huang et al. (2020), traffic density can be used to measure transport infrastructure, which was expressed as the ratio of the sum of road miles, railroad miles, and inland waterway miles to the land area of each province [2,5].

#### 4.2.2. High-Quality Development

##### Construction of the Indicator System

As China’s development approaches the new era, high-quality development is development that can meet the growing demand of people for a better life and is an important way to build a beautiful China, while following the new development concept of “innovation, coordination, green, openness, and sharing” [53]. This five-dimensional new development concept also indicates the direction, ideas, and focus underpinning China’s high-quality development. Based on the five-dimensional new development concept and the views of previous researchers [54], we argue that the five dimensions of innovation, coordination, sustainability, openness, and sharing should be the characteristics of high-quality development.

First, from the perspective of innovation, based on the fact that innovation is the source of power and potential, high-quality development can promote efficiency improvements through innovation, thus improving the quality of economic development. This study therefore measured three aspects of innovativeness: innovation input, innovation output, and efficiency improvement.

Second, from the perspective of coordination, China’s economic and social development has the problematic feature of uneven and insufficient regional development, which could be effectively solved by improving the efficiency of resource allocation and the rational use of resources, highlighting the importance of coordinated development. Coordination is the key to achieving high-quality regional co-development, and this study therefore measured three aspects of coordination: industrial coordination, urban–rural coordination, and regional coordination.

Third, from the perspective of sustainability, the new era of green development has led to the concept of “green mountains are golden mountains”. The rational use of natural resources is a prerequisite for sustainable economic development. Additionally, stable development can effectively restrict large fluctuations in prices, employment, and polluting industries, and is a tool to keep the national economy stable. Sustainable development is an important way to achieve high-quality development, and this study measured sustainability in terms of both green development and stable development.

Fourth, from the perspective of openness, alongside the implementation of policies such as the “One Belt, One Road” and “Free Trade Zone”, China has established cooperative relationships with many countries, and the level of openness has been continuously improved, which is also a necessary path to achieve high-quality development. This study measured three aspects of openness: foreign investment, foreign capital utilization, and foreign trade.

Finally, from the perspective of sharing, to realize the desire for a better and happier life among both urban and rural residents, to improve their quality of life, to meet their spiritual needs, and to ensure they receive the benefits of high-quality development, shareability is an important judgment criterion. This study measured five aspects of shareability: health, education, income, consumption, and leisure.

Considering the principles of high-quality development based on the new development concept, and by drawing on representative literature [12,54], an indicator system was developed following the principles of scientificity, dynamism, operability, and comprehensiveness. From the five aspects of innovation, coordination, openness, sustainability, and sharing, an evaluation index of China’s high-quality development was constructed that consisted of five primary indicators, 16 secondary indicators, and 39 tertiary indicators. The details are shown in Table 1.

##### Measurement of the Indicator System

This study developed a comprehensive inter-provincial high-quality development index for China for the period 2004–2017, with the details shown in Table 1. As can be seen from Table 1, the attributes of each indicator were different and the data needed to be dimensionless before measurements could be made. First, taking the inverse of the negative indicator and the inverse of the deviation of the moderate indicator, the positive indicator followed the principle of the larger the better. Second, on the basis of the above data processing, this study adopted the practices of previous researchers [53] and used the entropy weight method to determine a high-quality development index for China. The specific steps were as follows.

First, a comprehensive evaluation system of high-quality development was developed, and each indicator was processed to be dimensionless, with its value recorded as *W_j_*. The index level matrix *Y* = (*x_ij_*)*_m_*_× *n*_ was established after standardized processing. Second, the same metric was used to quantify the comprehensive indicators of high-quality development, and the value of the j item high-quality development indicator of each province in year *i* was calculated as *W_ij_*. Third, the entropy value of the j item high-quality development indicator *e_ij_* was calculated. The larger the *e_ij_*, the smaller the variability among the high-quality development indicators and the less important the indicators were, and vice versa. Fourth, the variability *d_j_* of the *j* item high-quality development indicator was calculated. Fifth, the weight of the j item quality development indicator was calculated. Finally, based on the measurement method of the United Nations Human Development Index (HDI), a final comprehensive score of high-quality development was obtained for each province. The specific equations are shown as (6)–(11). The index value was calculated according to Equation (10) after the dimensionless processing of each indicator in the comprehensive evaluation system of high-quality development. The comprehensive score of the high-quality development index (*P*) was also calculated for each province according to Equation (11). The value of *p* was between 0 and 1, and the closer it was to 1, the higher the quality of development of the province, and vice versa. Using the results of the entropy method, it was possible to compare the overall level of high-quality development of each province and also to observe the historical process of high-quality development in each province.

(6) $$Y=\left[\begin{array}{ccc} x_{11} & \cdots & x_{1n}\\ \vdots & \ddots & \vdots \\ x_{m1} & \cdots & x_{mn} \end{array}\right]$$

(7) $$Z_{ij}=x_{ij}/\sum x_{ij}$$

(8) $$e_{ij}=-k\sum z_{ij}\ln z_{ij},k=1/\ln m$$

(9) $$d_{j}=1-e_{j}$$

(10) $$h_{j}=d_{j}/\sum d_{j}$$

(11) $$P=\sum_{i=1}^{n}W_{j}h_{j}/\sum_{i=1}^{n}h_{j}$$

**Figure 3:**
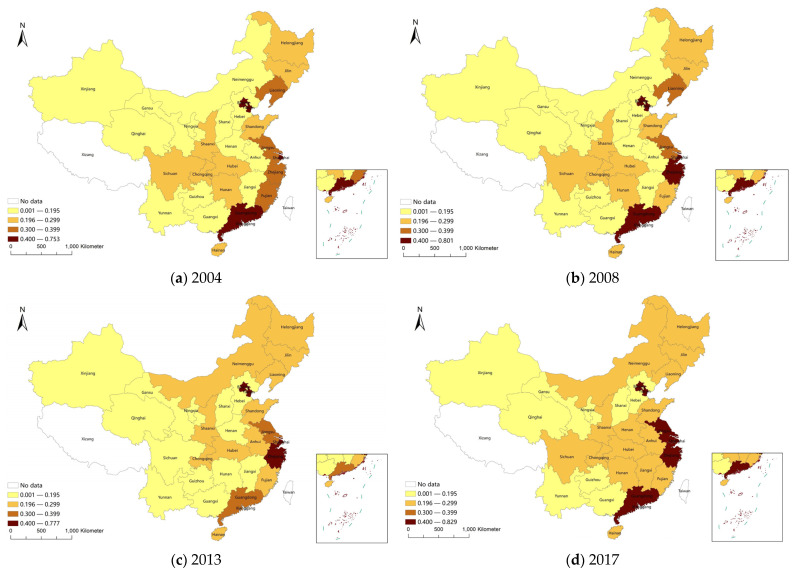
Composite index of high-quality development of Chinese provinces.

Based on the data availability, the comprehensive score of the high-quality development index was determined for 30 provinces in China for the period 2004–2017, with Tibet, Hong Kong, Macao, and Taiwan not included. The comprehensive scores of the high-quality development index of each province in selected years are shown in Table 2. The comprehensive scores of the high-quality development index of the 30 Chinese provinces in 2004, 2009, 2013, and 2017 were mapped and are shown in Figure 3. There was little change in the comprehensive score of the high-quality development index of each province over the period of 2004–2017. There were obvious differences in the comprehensive score of the high-quality development index among provinces, reflecting the unbalanced regional development throughout China. The index values for developed regions were very similar, with a cluster of high values in the eastern coastal region, and the development index of the less developed central and western regions being relatively low. Therefore, in the future, the government should accelerate the high-quality development of central and western regions, promote coordinated regional development, gradually narrow regional differences, and realize a new pattern of balanced development in eastern, central, and western regions.

#### 4.2.3. Industrial Pollution Emission Intensity

According to Sun et al. (2019), industrial pollution emission intensity mainly refers to pollutant emissions per unit of industrial value added [2]. Considering the availability of data in each province, in this study, industrial pollution emission intensity was determined by dividing industrial pollution emissions by the value added of the secondary industry, focusing on the impact of transport infrastructure and high-quality development on industrial sulfur dioxide emission intensity (SO_2_).

#### 4.2.4. Other Variables

Industrial agglomeration (AGG): there are many measures of industrial agglomeration in the existing literature, such as industrial concentration, the locational quotient method (LQM), the Ellison–Glaeser (E-G) index, and the Herfindahl–Hirschman index. Because the dependent variable was industrial pollution, the LQM of the secondary industry was used as a measure of industrial agglomeration [5]. $AGG_{it}=\left(q_{sit}/q_{it}\right)/\left(q_{snt}/q_{nt}\right)$, where $AGG_{it}$ represents the LQM of the secondary industry in the ith region in year t; $q_{sit}$ represents the value of the secondary industry in the ith region in year *t*; $q_{it}$ represents the total output value in the ith region in year *t*; $q_{snt}$ represents the value of the secondary industry in all regions of the country in year *t*; and $q_{nt}$ represents the national total output value in year *t*. This index was used to measure the degree of specialization of the local secondary industry. Compared with the national level, the larger the index value, the higher the degree of specialization of the local secondary industry, i.e., the higher the degree of industrial agglomeration.

Control variables: to minimize estimation bias due to omitted variables, reference was made to the existing literature on industrial pollution emissions [5] and the quality of economic development [5,55], and the following control variables were set. Environmental regulation (ER), which was measured as the amount of investment in environmental management as a proportion of GDP. Income level of workers (INC), which was measured as the per capita income of workers in employment. Technology development level (TEC), which was measured as the expenditure on science and technology. Population density (POP), which was measured as the number of people per unit area. Human capital level (EDU), which was measured as the number of university students per 10,000 people. Real estate (EST), which was measured as the proportion of real estate investment. Economic growth level (PERGDP), which was measured as GDP per capita. Urbanization level (URB), which was measured as the percentage of the population in urban areas. Energy intensity (ENER), which was measured as the contribution of energy consumption to overall GDP.

### 4.3. Data Sources

Due to the availability of data, this study considered 30 Chinese provinces (excluding Tibet, Hong Kong, Macao, and Taiwan) spanning 14 years from 2004 to 2017, and the research data were obtained from the China Statistical Yearbook, China Industrial Statistical Yearbook, China Environment Yearbook, China Energy Statistical Yearbook, China Labor Statistical Yearbook, China Science and Technology Statistical Yearbook, China Fixed Assets Statistical Yearbook, and the statistical yearbooks of the 30 provinces, as well as the National Bureau of Statistics, China Economic and Social Big Data Research Platform, CENSUS Statistical Database, and Wind Database. The data descriptions and descriptions of the variables referred to above, core explanatory variables, and a series of control variables are given in Table 3.

## 5. Results and Discussion

### 5.1. Baseline Results

Table 4 shows the baseline results for Equations (1) and (2). We select the appropriate model based on the F-test and the Hausman test, reported in the last two rows. The tests indicate that the fixed effect model is appropriate in most models. Table 4 gives the results of the benchmark regressions for models (1)–(3). The results showed that transport infrastructure was significantly positively associated with industrial pollution and the secondary term of high-quality development was significantly negatively associated with industrial pollution after controlling for a series of province-specific variables as well as fixed time effects. This indicated that transport infrastructure promoted industrial pollution emissions, while the relationship between high-quality development and industrial pollution had an inverted U-shaped relationship, i.e., as high-quality development increased, it initially promoted industrial pollution emissions and then suppressed industrial pollution emissions after reaching an inflection point. This was because in the early stage of high-quality development, the increase in production efficiency of enterprises is accompanied by an increase in related costs, and therefore, enterprises continue to increase the price of products or the production speed and increase the production output of existing products. In the early stage of high-quality development, the environmental standards are low, environmental legislation and regulations are not perfect, and environmental protection technology is weak or even absent, which results in high industrial SO_2_ emissions, leading to a further deterioration in environmental quality. When the quality of economic development improves to a certain level, it will lead to further technological improvements and the optimization of production level and industrial structure. At this point enterprise production efficiency is substantially improved, costs are reduced, environmental protection technology matures, and environmental legislation and regulations are perfected; thus, improving resource utilization and environmental quality. From the absolute values of the basic regression coefficients in Table 4, high-quality development had a much greater value than transport infrastructure, indicating that high-quality development had a strong effect on reducing industrial pollution. From the regression results of the control variables, it was found that the level of scientific and technological development was significantly negatively correlated with industrial pollution emissions, indicating that an improvement in the level of scientific and technological development can effectively suppress industrial pollution emissions. The level of human capital was significantly positively correlated with industrial pollution, indicating that an improvement in the level of human capital can promote industrial pollution emissions.

Economic and social development in China is still unbalanced and insufficient, and the regional imbalance is particularly obvious when observed on a line graph of the high-quality development index. The effect of the heterogeneity between transport infrastructure and high-quality development on industrial pollution emissions was further evaluated by defining three major regions: eastern, central, and western regions. Columns (1), (2), and (3) of Table 5 show the regression results of the effects of transport infrastructure and high-quality development on industrial pollution emissions in the three different regions, respectively. The regression results for transport infrastructure showed that transport infrastructure in the eastern region was significantly and positively correlated with industrial pollution emissions, and the estimated coefficients were larger than those of the national sample, indicating that transport infrastructure had a more significant impact on industrial pollution emissions in the eastern region. The traffic infrastructure in the central region was not significantly related to industrial pollution emissions, indicating a lower degree of influence. Transport infrastructure in the western region was significantly and positively correlated with industrial pollution emissions, and the estimated coefficients were larger than the national sample, indicating that transport infrastructure had a more significant impact on industrial pollution emissions in the western region. Thus, H1 was verified.

From the regression results of high-quality development shown in Table 5, the primary term of high-quality development in the eastern region was significantly positively correlated with industrial pollution emissions, and the secondary term of high-quality development was significantly negatively correlated with industrial pollution emissions, confirming that high-quality development significantly increased industrial pollution emissions in the early stage, and an inflection point occurred after reaching a certain development stage, after which industrial pollution emissions were significantly suppressed. In the central region, the trend of the impact of high-quality development on industrial pollution emissions was similar to that of the east, but it was not significant, indicating a lower degree of impact. In the western region, the primary term of high-quality development was significantly positively correlated with industrial pollution emissions, and the secondary term of high-quality development was significantly negatively correlated with industrial pollution emissions, confirming that high-quality development significantly increased industrial pollution emissions in the early stage, and after reaching a certain stage of development, there was an inflection point after which industrial pollution emissions were significantly suppressed. The degree of influence was higher than that in the eastern and central regions. This was largely because the eastern region is more developed, and the effect of high-quality development on industrial pollution emission reduction was obvious. The central region is in the middle stage of industrialization, with a prominent “late-stage disadvantage” in economic development. Local governments pay more attention to the new normal of economic development at this stage, which is characterized by stronger environmental constraints and a reduction in the economic growth rate [56]. In this region, high-quality development has not yet significantly affected industrial pollution emission reduction. The western region is more undeveloped and is currently in a stage of vigorous economic development, but the effect of high-quality development on industrial pollution reduction was obvious. Thus, H2 was verified.

### 5.2. Endogeneity Issues and Instrumental Variables

Although the model results suggested that transport infrastructure can increase industrial pollution emissions, and high-quality development helps promote reductions in industrial pollution emissions, the results may still be biased due to endogeneity problems. On the one hand, the better the effect of industrial pollution emission reduction, the better the quality of the transport infrastructure network and the better the quality of the economy and society will be. There may be a two-way causal relationship between transport infrastructure, high-quality development, and industrial pollution. However, it is difficult to enumerate the individual factors affecting industrial pollution, and there will inevitably be omitted variables. To avoid any bias in the model results caused by endogeneity and omitted variables, reference was made to Shi et al. (2020), and the lagged terms of the indexes of transport infrastructure and high-quality development were used as the instrumental variables of both to correct the bias of the estimation results [55]. Transport infrastructure and high-quality development had intertemporal effects on industrial pollution, and in the prior period, transport infrastructure and high-quality development indices were not affected by the later period industrial pollution emissions. Therefore, lagging the transport infrastructure and high-quality development indexes could effectively address the two-way causality between them. For this reason, the regression results of the transport infrastructure and high-quality development indices are shown in Table 6, with one and two lagged periods, respectively. The results showed that the estimated coefficients of transport infrastructure and high-quality development were still statistically significant, which again verified that transport infrastructure could significantly increase industrial pollution emissions, while high-quality development could significantly suppress industrial pollution emissions.

Columns (1)–(6) of Table 7 show the estimated results of the impact of transport infrastructure and high-quality development on industrial pollution emissions for different regions with one and two lags and with significant overall lagged effects. Transport infrastructure in the eastern region was significantly and positively correlated with industrial pollution emissions, and the correlation coefficients were larger than those of the national sample, indicating that transport infrastructure had a more significant impact on industrial pollution emissions in the eastern region. The traffic infrastructure in the central region was significantly negatively correlated with industrial pollution emissions, indicating that traffic infrastructure had a lagging effect on industrial pollution. Increasing the construction of traffic infrastructure in the central region would be conducive to the reduction in industrial pollution, probably because the central region is an important hub linking the eastern and western regions. If its transportation network of roads, railroads, and water transport was more extensive, the economic hinterland of the eastern region would be both expanded and improved, which would also improve the accessibility of the western region. The correlation coefficient was larger than that of the national sample, indicating that the most significant impact of transport infrastructure on industrial pollution emissions occurred in the western region. From the regression results for high-quality development, the primary term of high-quality development in the eastern region was significantly positively correlated with industrial pollution emissions, and the secondary term of high-quality development was significantly negatively correlated with industrial pollution emissions. The trend of the impact of high-quality development on industrial pollution emissions in the central region was similar to that in the eastern region, but it was not significant, indicating a lower degree of impact. The primary term of high-quality development in the western region was significantly positively correlated with industrial pollution emissions, and the secondary term of high-quality development was significantly negatively correlated with industrial pollution emissions. This indicated that high-quality development significantly increased industrial pollution emissions in the early stage of development, until eventually an inflection point was reached where industrial pollution emissions were significantly suppressed, and the degree of influence was higher than that in the eastern and central regions.

### 5.3. Mechanism Identification: Industrial Agglomeration

Previous studies empirically tested the effects of transport infrastructure and high-quality development on industrial pollution emissions. In this study, the mechanism of the impact of transport infrastructure on high-quality development was investigated using a mediating effect model to test whether industrial agglomeration was the transmission path. Table 8 presents the results of the mediating effects model. Column 1 presents the results with industrial agglomeration as the dependent variable, indicating a positive and significant relationship between transport infrastructure and industrial agglomeration (β = 0.000586, *p* < 0.01). In columns 2 and 3, the coefficient of transport infrastructure can be seen to decrease significantly (dropping from −0.000148 to −0.000143). The coefficient of industrial agglomeration was also significant (β = −0.0852, *p* < 0.01), which suggests that industrial agglomeration (AGG) plays a role in the association between transport infrastructure and high-quality development. This suggests that industrial agglomeration (AGG) plays a mediating role in the association between transport infrastructure and high-quality development, and TRA reduces the inhibitory effect on high-quality development by promoting industrial agglomeration.

In columns 2 and 3 of Table 8, the coefficient of transport infrastructure can be seen to decrease significantly (dropping from 0.00183 to 0.00155), and the coefficient of industrial agglomeration was also significant (β = −1.704, *p* < 0.01). This suggests that industrial agglomeration (AGG) played a mediating role in the association between transport infrastructure construction and industrial pollution emissions, i.e., TRA reduces industrial SO_2_ emissions by promoting industrial agglomeration. Thus, H3 was verified.

### 5.4. Robustness Test

The aforementioned instrumental variables were used as the only core explanatory variables, with a related concern being that the control variables may also have endogeneity problems caused by “reverse causality”. The reliability of the conclusions was further ensured. In addition, tailoring was applied to eliminate the sample outliers, with column 4 in Table 9 showing the results after a 5% tailoring of the sample. The results after eliminating the outliers were basically consistent with the baseline regression findings. Therefore, the robustness of the study findings was considered to be good.

## 6. Conclusions

### 6.1. Conclusions

China’s ongoing modernization is based on a new development stage, the implementation of a new development concept, and the building of a new development pattern. The Fifth Plenary Session of the 19th CPC Central Committee and the Central Economic Work Conference made important arrangements for ecological protection, continued to promote pollution prevention and control, increased the optimization and adjustment of transportation structure, and promoted public-to-rail, public-to-water, and multimodal transportation. Therefore, promoting high-quality development is an active choice to adapt to the new normal of economic development and is the way to build a modern economic system. Based on China’s new development concept, this study developed a high-quality development index for China that consisted of 5 primary indicators, 16 secondary indicators, and 39 tertiary indicators based on the five aspects of innovation, coordination, openness, sustainability, and sharing. The index used the entropy weight method to measure the comprehensive score of the high-quality development index for 30 Chinese provinces (excluding Tibet, Hong Kong, Macao, and Taiwan). Based on the data for these 30 provinces covering the 14 years from 2004 to 2017, an empirical study was conducted to investigate the mechanism by which transport infrastructure and high-quality development affected industrial pollution emissions, and heterogeneity tests were conducted in three major regions: the eastern, central, and western regions. The regression results revealed several features.

First, the construction of transport infrastructure significantly promoted industrial pollution emissions, and there was a regional heterogeneity and time lag, with a significant positive correlation in the eastern and western regions and a significant negative correlation in the central region.

Second, the relationship between high-quality development and industrial pollution had an inverted “U” shape, and there was both regional heterogeneity and a time lag, with a significant positive correlation in the eastern and western regions, and no significant correlation in the central region.

Third, transport infrastructure significantly affected high-quality development and industrial pollution through industrial agglomeration, and transport infrastructure reduced the inhibitory effect on high-quality development by promoting industrial agglomeration. Transport infrastructure also reduced industrial SO_2_ emissions by promoting industrial agglomeration.

Fourth, improvements in the level of scientific and technological development could suppress industrial pollution emissions, while improvements in the level of human capital could promote industrial pollution emissions.

### 6.2. Policy Recommendations

Based on the research findings, several policy recommendations can be made.

First, environmental factors should be incorporated into the transport infrastructure planning process. The transport infrastructure network should be optimized by region, with a focus on building green transport infrastructure construction to effectively improve the ecological environment. For the eastern region, there should be a focus on the transformation of highways and railroads, as well as the construction of inland waterways, while ensuring a transition from quantity to quality. Clean energy generation should be implemented and a green transportation network should be established to alleviate industrial pollution. For the central region, transportation infrastructure should be seen as a link, playing the role of radiating urban and economic development to the surrounding areas. The construction of green and intelligent transportation network systems should be encouraged, and the coordinated development of the regional economy should be promoted. For the western region, policy support should be increased, the upgrading of transport infrastructure networks should be accelerated, and the construction of high-speed railroads, highways, and other infrastructure is required. The overall outcome should be a clean and efficient transportation system.

Second, high-quality development should be promoted, with a new balance between economic development and environmental quality, which should be maintained by combining high-quality development and environmental protection. Exchanges between the western region and the central and eastern regions should be strengthened to steadily achieve high-quality regional economic development and effectively protect the ecological environment.

Third, industrial agglomeration and the achievement of a reasonable industrial layout should be promoted to establish the regional advantages of the industrial development pattern, encourage enterprises to innovate and upgrade, facilitate green production, and promote the positive role of transport infrastructure on high-quality development and industrial pollution.

Fourth, there is a need to improve the level of scientific and technological development to encourage innovation in industrial pollution reduction. At the same time, there is a need to build green human capital, stimulate a new green energy dynamic, gradually optimize the industrial and economic structure, and improve the natural environment.

### 6.3. Limitations

Our work has examined the impact of transport infrastructure, and high-quality development on industrial pollution emissions from a fresh perspective and has led to some interesting conclusions, but there is still some work to be done. Firstly, this study is limited to data on SO_2_ emissions, and indeed other air pollutants such as fine particulate matter (PM2.5) and nitric oxide (NO), and our work should be further extended to assess emissions of other pollutants to confirm the heterogeneous impact of transport infrastructure and high-quality development. Secondly, this study focuses on the relationship between transport infrastructure and the output of polluting activities within Chinese provinces, while ignoring the spatial spillover and spatial unit autocorrelation of transport infrastructure in other provinces, so the impact of transport infrastructure on pollution emissions between Chinese provinces should be studied in the future.

## Figures and Tables

**Table 1:** Indicator system for evaluating quality development in China.

Primary	Secondary	Basic Indicators	Unit	Attribute
Innovation	Innovation input	Number of R&D personnel/Number of All Practitioners	%	Positive
		R&D expenditure of R&D institutions/GDP	%	Positive
		The average number of years of limited education for employees in each province	year/person	Positive
	Innovation output	Number of domestic patent applications granted per capita	pieces/million	Positive
		Technology Market Turnover/GDP	%	Positive
	Efficiency improvement	GDP/Number of All Practitioners	%	Positive
		GDP/Total social fixed asset investment	10,000 RMB/person	Positive
		GDP/Tons of standard coal	10,000 RMB/ton	Positive
		Total Grain Production/Total arable land area	ten tons/hectare	Positive
		Domestic budget funds/Total social fixed asset investment	%	Negative
		Government consumption expenditure as a share of total consumption expenditure	%	Negative
		The proportion of fixed asset investment in non-state economy	%	Positive
		Number of Individuals Employed/Number of All Practitioners	%	Positive
		Financial sector value added/GDP	%	Positive
Coordination	Industry Coordination	The inverse of Theil index	-	Positive
		Tertiary industry value/Secondary industry value	%	Positive
	Urban-rural coordination	Per capita income of urban residents/Per capita income of rural residents	%	Moderate
		Per capita consumption of urban residents/Per capita consumption of rural residents	%	Moderate
	Regional Coordination	GDP per capita by province/National GDP per capita	%	Positive
		Consumption level of residents by province/National average consumption level	%	Positive
Openness	Foreign Investment	OFDI/GDP	%	Positive
	Utilization of foreign investment	FDI/GDP	%	Positive
	Foreign Trade	Total import and export/GDP	%	Positive
Sustainability	Green Development	Greening coverage of built-up areas	%	Positive
		Nature Reserve Area/Area of jurisdiction	%	Positive
		Forest cover	%	Positive
		SO_2_ emission/GDP	tons/million	Negative
		Wastewater discharge/GDP	tons/million	Negative
		General industrial solid waste generation/GDP	tons/million	Negative
		Environmental pollution control investment/GDP	%	Negative
	Stable development	Urban registered unemployment rate	%	Negative
		Industrial producer ex-factory price index	%	Positive
		Consumer Price Index	%	Positive
		Regional GDP growth rate	%	Positive
Sharing	Health	Number of beds in medical and health institutions per unit of population	pcs/person	Positive
	Education	Expenditure on education as a share of fiscal expenditure	%	Positive
	Revenue	Average wage of employees in employment	RMB/person	Positive
	Consumption	Per capita consumption expenditure	RMB/person	Positive
	Leisure	Engel’s coefficient of resident travel	-	Positive

Note: “Positive (Negative)” in the “Attributes” column of the indicator indicates that the measure is a positive (negative) indicator under the set measurement approach, the larger (smaller) the better.

**Table 2:** Composite index of high-quality development in China, 2004–2017.

Province	2004	2005	2007	2009	2011	2013	2015	2017
Beijing	0.754	0.809	0.798	0.786	0.779	0.778	0.784	0.829
Tianjin	0.439	0.481	0.445	0.427	0.413	0.425	0.436	0.435
Hebei	0.171	0.186	0.164	0.196	0.186	0.176	0.190	0.192
Shanxi	0.157	0.161	0.171	0.182	0.187	0.187	0.188	0.195
Neimenggu	0.177	0.196	0.169	0.182	0.214	0.224	0.207	0.200
Liaoning	0.317	0.315	0.275	0.297	0.288	0.296	0.259	0.295
Jilin	0.234	0.236	0.219	0.211	0.207	0.207	0.206	0.212
Heilongjiang	0.209	0.217	0.198	0.224	0.197	0.213	0.235	0.225
Shanghai	0.662	0.683	0.690	0.687	0.636	0.596	0.609	0.643
Jiangsu	0.329	0.368	0.348	0.373	0.393	0.390	0.386	0.404
Zhejiang	0.366	0.404	0.404	0.433	0.427	0.407	0.397	0.423
Anhui	0.169	0.162	0.160	0.187	0.197	0.201	0.204	0.222
Fujian	0.312	0.289	0.278	0.288	0.278	0.275	0.270	0.285
Jiangxi	0.194	0.193	0.174	0.194	0.199	0.193	0.195	0.217
Shandong	0.251	0.278	0.255	0.271	0.255	0.260	0.255	0.277
Henan	0.154	0.170	0.157	0.166	0.159	0.160	0.170	0.200
Hubei	0.201	0.208	0.189	0.201	0.194	0.208	0.231	0.257
Hunan	0.202	0.209	0.185	0.211	0.181	0.188	0.208	0.223
Guangdong	0.449	0.476	0.460	0.436	0.395	0.388	0.362	0.400
Guangxi	0.157	0.163	0.152	0.182	0.159	0.164	0.155	0.170
Hainan	0.251	0.214	0.222	0.232	0.245	0.250	0.261	0.264
Chongqing	0.233	0.239	0.192	0.221	0.245	0.234	0.244	0.266
Sichuan	0.201	0.198	0.182	0.184	0.188	0.192	0.198	0.227
Guizhou	0.096	0.103	0.093	0.103	0.117	0.129	0.142	0.166
Yunnan	0.147	0.147	0.128	0.161	0.161	0.161	0.148	0.162
Shaanxi	0.198	0.196	0.178	0.195	0.212	0.222	0.235	0.233
Gansu	0.142	0.150	0.151	0.153	0.129	0.157	0.173	0.169
Qinghai	0.184	0.169	0.146	0.151	0.130	0.130	0.157	0.161
Ningxia	0.176	0.158	0.169	0.180	0.160	0.158	0.179	0.187
Xinjiang	0.194	0.195	0.175	0.209	0.169	0.171	0.204	0.188

**Table 3:** Descriptive statistics of the variables.

Variables	Definition	Obs.	Mean	S. D	Min	Max	Unit
SO_2_	Industrial SO_2_ emission intensity	420	128.0613	138.5101	0.7132	831.1027	tons/billion
TRA	Traffic density	420	95.6716	64.7235	5.4699	398.8711	km/100 km^2^
HQD	High-quality development	420	0.2564	0.1469	0.0859	0.8359	-
AGG	Industrial clustering	420	0.9741	0.1595	0.4447	1.2279	%
ER	Environmental regulation	420	1.3462	0.6675	0.3000	4.2400	%
INC	Employee income levels	420	0.4670	0.0808	0.1901	0.6148	RMB/person
TEC	Level of technological development	420	63.3104	95.4316	0.4200	823.8900	billion
POP	Population density	420	450.3734	637.5155	11.9854	3826.1970	person/km^2^
EDU	Human capital levels	420	166.7550	69.2046	17.8897	348.9753	%
EST	Real estate investment	420	0.1891	0.1039	0.0613	0.5827	%
PERGDP	Economic growth	420	10.2937	0.6784	8.3133	11.7680	10,000 RMB/person
URB	Level of urbanization	420	52.0868	14.1810	13.8850	89.6066	%
ENER	Energy intensity	420	1.1006	0.6434	0.2530	4.3232	tons of standard coal per 10,000 RMB

**Table 4:** Transportation infrastructure, high-quality development, and industrial pollution: baseline regression results.

Dependent Variable	(1)	(2)	(3)
	SO_2_	SO_2_	SO_2_
TRA	0.00183 **		0.00181 **
	(0.0009)		(0.0009)
HQD^2^		−3.195 ***	−3.275 ***
		(1.2302)	(1.2252)
HQD		1.251	1.375
		(1.1756)	(1.1717)
ER	0.0471	0.0509	0.0549 *
	(0.0314)	(0.0313)	(0.0312)
INC	−0.43	−0.457	−0.393
	(0.2798)	(0.2817)	(0.2821)
TEC	−0.281 ***	−0.248 ***	−0.286 ***
	(0.0593)	(0.0569)	(0.0595)
POP	−0.214	−0.45	−0.377
	(0.3986)	(0.4003)	(0.4000)
EDU	0.274 **	0.275 **	0.282 ***
	(0.1073)	(0.1064)	(0.1059)
EST	−0.0857	−0.255	−0.291
	(0.3872)	(0.3894)	(0.3880)
constant	9.666 **	11.26 ***	10.13 **
	(3.9975)	(3.9289)	(3.9479)
Year fixed effect	Y	Y	Y
Province fixed effect	Y	Y	Y
N	420	420	420
R^2^_a	0.908	0.9096	0.9104
F	32.05 ***	18.60 ***	18.56 ***
Hausman	29.96 ***	43.57 ***	42.83 ***

Note: Standard errors are presented in the parentheses. *** indicates *p* < 0.01. ** indicates *p* < 0.05. * indicates *p* < 0.1.

**Table 5:** Regional heterogeneity test.

Dependent Variable	(1)	(2)	(2)
	East	Central	West
	SO_2_	SO_2_	SO_2_
TRA	0.00431 *	−0.00321	0.00348 *
	(0.0024)	(0.0033)	(0.0016)
HQD^2^	−3.1988 **	−16.12	−50.20 **
	(1.2911)	(58.6760)	(20.1424)
HQD	2.2380 *	7.671	20.87 **
	(1.2018)	(23.5823)	(8.2986)
ER	−0.0245	0.0186	0.0729
	(0.0711)	(0.0494)	(0.0492)
INC	0.186	−0.522	−0.257
	(0.7100)	(1.0811)	(0.7188)
TEC	−0.0869	−0.206	−0.756 ***
	(0.0862) *	(0.1327)	(0.1756)
POP	−0.266	−1.162	3.731 **
	(0.1681)	(2.8317)	(1.3680)
EDU	0.104	−0.151	0.419 ***
	(0.2608)	(0.4269)	(0.1020)
EST	−0.0357	−2.084	3.634 **
	(0.6504)	(1.6222)	(1.2190)
constant	5.762	16.91	−13.79
	(5.2613)	(17.4356)	(11.8126)
Year fixed effect	Y	Y	Y
Province fixed effect	Y	Y	Y
N	168	126	126
R^2^_a	0.9318	0.9536	0.9132

Note: Standard errors are presented in the parentheses. *** indicates *p* < 0.01. ** indicates *p* < 0.05. * indicates *p* < 0.1.

**Table 6:** Instrumental variable method (2SLS) estimation results.

Dependent Variable	IV_2SLS (One-Phase Lag)			IV_2SLS (Two-Phase Lag)		
	(1)	(2)	(3)	(4)	(5)	(6)
	SO_2_	SO_2_	SO_2_	SO_2_	SO_2_	SO_2_
TRA	0.00197 *		0.00197 *	0.00424 ***		0.00417 ***
	(0.001)		(0.001)	(0.0015)		(0.0015)
HQD^2^		−3.438 ***	−3.476 ***		−3.653 ***	−3.541 ***
		(1.2168)	(1.2122)		(1.2331)	(1.2206)
HQD		1.555	1.615		1.755	1.627
		(1.1764)	(1.1722)		(1.1996)	(1.1877)
ER	0.0512	0.0547 *	0.0589 *	0.0433	0.0432	0.0509
	(0.0319)	(0.0316)	(0.0316)	(0.0328)	(0.0325)	(0.0323)
INC	−0.458	−0.479	−0.421	−0.378	−0.408	−0.325
	(0.3047)	(0.3073)	(0.3076)	(0.3295)	(0.3333)	(0.3311)
TEC	−0.261 ***	−0.238 ***	−0.270 ***	−0.267 ***	−0.234 ***	−0.278 ***
	(0.0619)	(0.0598)	(0.0619)	(0.0669)	(0.0655)	(0.0667)
POP	−0.214	−0.454	−0.432	−0.376	−0.39	−0.515
	(0.4363)	(0.4399)	(0.4383)	(0.4933)	(0.4944)	(0.4912)
EDU	0.303 ***	0.313 ***	0.312 ***	0.384 ***	0.405 ***	0.384 ***
	(0.1098)	(0.1086)	(0.1082)	(0.1161)	(0.1151)	(0.1141)
EST	−0.0806	−0.241	−0.282	−0.199	−0.318	−0.423
	(0.3994)	(0.3997)	(0.3987)	(0.4195)	(0.4204)	(0.4176)
constant	9.809 **	11.31 ***	10.56 **	9.206 *	9.702 **	9.400 **
	(4.3352)	(4.2759)	(4.2774)	(4.7377)	(4.7065)	(4.6575)
Year fixed effect	Y	Y	Y	Y	Y	Y
Province fixed effect	Y	Y	Y	Y	Y	Y
N	390	390	390	360	360	360

Note: Standard errors are presented in the parentheses. *** indicates *p* < 0.01. ** indicates *p* < 0.05. * indicates *p* < 0.1.

**Table 7:** Regional heterogeneity test: instrumental variable method (2SLS) estimation results.

Dependent Variable	IV_2SLS (One-Phase Lag)			IV_2SLS (Two-Phase Lag)		
	(1)	(2)	(3)	(4)	(5)	(6)
	East	Central	West	East	Central	West
	SO_2_	SO_2_	SO_2_	SO_2_	SO_2_	SO_2_
TRA	0.00634 **	−0.00439 **	0.00275 **	0.0156 ***	−0.0147 ***	0.00467 **
	(0.0028)	(0.0022)	(0.0014)	(0.0052)	(0.0049)	(0.0019)
HQD^2^	−3.932 **	−7.89	−47.23 ***	−4.183 **	18.39	−39.57 **
	(1.7629)	(34.2846)	(18.2133)	(1.8102)	(36.2433)	(18.7601)
HQD	3.391 *	3.906	19.56 **	3.751 *	−7.076	16.08 **
	(1.9832)	(14.3015)	(7.8543)	(2.0527)	(15.1471)	(8.125)
ER	0.0344	0.0344	0.0778	−0.0565	0.0345	0.0828
	(0.057)	(0.057)	(0.0561)	(0.0602)	(0.0556)	(0.0574)
INC	−0.352	−0.613	−0.307	−0.302	−1.079	−0.0232
	(0.4715)	(0.6127)	(0.7326)	(0.5469)	(0.6913)	(0.7897)
TEC	−0.113	−0.156 *	−0.690 ***	−0.225 *	0.00956	−0.665 ***
	(0.1071)	(0.0908)	(0.1598)	(0.1176)	(0.1096)	(0.1782)
POP	−1.564 **	−0.49	2.592 *	−2.142 ***	1.469	1.251
	(0.6146)	(1.3091)	(1.4184)	(0.7058)	(1.5153)	(1.6635)
EDU	−0.152	−0.0338	0.435 ***	−0.262	−0.152	0.487 ***
	(0.2184)	(0.2824)	(0.1668)	(0.2477)	(0.3235)	(0.1704)
EST	−0.299	−2.096 *	3.687 ***	−0.871	−2.289 **	3.474 ***
	(0.501)	(1.0761)	(1.2718)	(0.5456)	(1.1378)	(1.3072)
constant	14.32	14.32	−7.759	21.01 ***	11.05	−4.264
	(9.4124)	(9.4124)	(11.2694)	(6.9335)	(9.6946)	(12.625)
Year fixed effect	Y	Y	Y	Y	Y	Y
Province fixed effect	Y	Y	Y	Y	Y	Y
N	117	117	117	144	108	108

Note: Standard errors are presented in the parentheses. *** indicates *p* < 0.01. ** indicates *p* < 0.05. * indicates *p* < 0.1.

**Table 8:** Analysis of the impact mechanism of transportation infrastructure on high-quality development and industrial pollution.

Dependent Variable	(1)	(2)	(3)	(4)	(5)
	AGG	HQD	HQD	SO_2_	SO_2_
TRA	0.000586 ***	−0.000148 **	−0.000143 *	0.00183 **	0.00155 *
	(0.0002)	(0.0001)	(0.0001)	(0.0009)	(0.0008)
AGG			−0.0852 ***		−1.704 ***
			(0.0204)		(0.1938)
TEC		0.00912	0.0115 **	−0.281 ***	
		(0.0056)	(0.0055)	(0.0593)	
POP	−0.387 ***	−0.103 ***	−0.118 ***	−0.214	−0.997 ***
	(0.1019)	(0.0349)	(0.0343)	(0.3986)	(0.3749)
EDU	0.0892 ***	−0.00481	−0.00094	0.274 **	0.487 ***
	(0.0272)	(0.0092)	(0.0090)	(0.1073)	(0.1028)
ENER	0.0236	−0.00545	0.00141		
	(0.0189)	(0.0068)	(0.0069)		
EST				−0.0857	−0.683 *
				(0.3872)	(0.3717)
PERGDP		0.0539 ***	0.0933 ***		
		(0.0160)	(0.0182)		
ER				0.0471	0.0419
				(0.0314)	(0.0295)
INC	0.305 ***			−0.43	−0.302
	(0.0708)			(0.2798)	(0.2598)
URB		0.00031	0.000311		
		(0.0003)	(0.0003)		
constant	−0.601	0.273	−0.000286	9.369 **	12.20 ***
	(1.0642)	(0.2973)	(0.2982)	(4.2011)	(3.8690)
Year fixed effect	Y	Y	Y	Y	Y
Province fixed effect	Y	Y	Y	Y	Y
N	420	420	420	420	420
R^2^_a	0.8276	0.9778	0.9788	0.9434	0.9504

Note: Standard errors are presented in the parentheses. *** indicates *p* < 0.01. ** indicates *p* < 0.05. * indicates *p* < 0.1.

**Table 9:** Robustness tests.

Dependent Variable	IV_2SLS (One-Phase Lag)			Remove Outliers
	(1)	(2)	(3)	(4)
	SO_2_	SO_2_	SO_2_	SO_2_
TRA	0.00189 *		0.00187 *	0.00143 *
	(0.0010)		(0.0010)	(0.0009)
HQD^2^		−3.440 ***	−3.471 ***	−2.382 **
		(1.2132)	(1.2093)	(1.1240)
HQD		1.749	1.813	1.038
		(1.1802)	(1.1769)	(1.0867)
ER	0.0291	0.0305	0.0352	0.0576 *
	(0.0320)	(0.0317)	(0.0317)	(0.0299)
INC	−0.583 **	−0.605 **	−0.560 *	−0.401
	(0.2862)	(0.2912)	(0.2913)	(0.2705)
TEC	−0.248 ***	−0.227 ***	−0.260 ***	−0.293 ***
	(0.0636)	(0.0609)	(0.0633)	(0.0573)
POP	−0.193	−0.375	−0.332	−0.182
	(0.4137)	(0.4150)	(0.4143)	(0.3850)
EDU	0.255 **	0.258 **	0.258 **	0.246 **
	(0.1107)	(0.1096)	(0.1092)	(0.1017)
EST	−0.567	−0.614	−0.646	−0.314
	(0.4173)	(0.4133)	(0.4123)	(0.3725)
constant	11.17 ***	12.32 ***	11.57 ***	9.327 **
	(4.1389)	(4.0799)	(4.0879)	(3.7904)
Year fixed effect	Y	Y	Y	Y
Province fixed effect	Y	Y	Y	Y
N	390	390	390	420

Note: Standard errors are presented in the parentheses. *** indicates *p* < 0.01. ** indicates *p* < 0.05. * indicates *p* < 0.1.

## Data Availability

Not applicable.
